# Supplementary material for: Complexome profiling of the Chlamydomonas psb28 mutant reveals TEF5 as an early PSII assembly factor
Source: Plant Cell. 2025 Mar 17;37(6):koaf055. doi: 10.1093/plcell/koaf055 (PMC12164590; doi:10.1093/plcell/koaf055)
Supplement: koaf055_Supplementary_Data [file koaf055_supplementary_data.zip › psb28_interactive.zip!/plots/intensity/qualityPlots/peptides/CHLM_Cre12.g498550.t1.1.html]

### Cre12.g498550.t1.1

Trivial name: CHLM  
SeqCoverage [median]: 54.90  
Mass: 34.99 kDa  
  
 

| Sequence | MissedCleavage | Mass [Da] | wtIntensity | muIntensity | Score |
| --- | --- | --- | --- | --- | --- |
| AYLHREEDVEAALK | 1 | 1642.83 | 545.56 | 2628.04 | 95.40 |
| AYLHREEDVEAALKR | 2 | 1798.93 | 14835.60 | 12455.11 | 151.74 |
| DYFNTAGFER | 0 | 1218.53 | 0.00 | 4896.85 | 98.04 |
| EEDVEAALK | 0 | 1002.49 | 0.00 | 759.89 | 52.72 |
| EEDVEAALKR | 1 | 1158.59 | 2418.64 | 5020.62 | 52.28 |
| EMTATSFYFSR | 0 | 1338.59 | 17341.77 | 38016.72 | 129.85 |
| FEALDLESVK | 0 | 1149.59 | 22771.72 | 55244.23 | 156.35 |
| FEALDLESVKGK | 1 | 1334.71 | 740.42 | 0.00 | 50.35 |
| GAAVSASDISAAMASEAEQR | 0 | 1920.88 | 33606.64 | 39989.20 | 184.15 |
| IGELFPGPSK | 0 | 1043.57 | 39787.66 | 57354.74 | 142.98 |
| IGELFPGPSKATR | 1 | 1371.75 | 0.00 | 783.19 | 57.02 |
| IYGETDEVNK | 0 | 1166.55 | 0.00 | 3145.11 | 113.71 |
| IYGETDEVNKVQLDIR | 1 | 1890.97 | 7430.09 | 6868.49 | 93.51 |
| KIYGETDEVNKVQLDIR | 2 | 2019.06 | 3688.20 | 3300.73 | 52.03 |
| LIISFAPK | 0 | 887.55 | 23737.85 | 50957.06 | 116.54 |
| LLEAIRE | 1 | 842.49 | 47835.82 | 56580.33 | 139.31 |
| REMTATSFYFSR | 1 | 1494.69 | 4465.75 | 5984.47 | 120.84 |
| RIGELFPGPSK | 1 | 1199.67 | 1553.34 | 2113.62 | 52.69 |
| SFDGDEK | 0 | 796.32 | 0.00 | 430.77 | 91.91 |
| SFDGDEKEAVKDYFNTAGFER | 2 | 2424.09 | 0.00 | 2099.83 | 43.70 |
| TGHAQTVDKVLR | 1 | 1323.73 | 4261.34 | 6154.37 | 185.57 |
| TLSYSILK | 0 | 923.53 | 3865.76 | 20309.30 | 89.14 |
| VAPKFEALDLESVK | 1 | 1544.85 | 12015.59 | 10876.30 | 82.91 |
| VAPKFEALDLESVKGK | 2 | 1729.96 | 559.15 | 627.77 | 30.54 |
| VDAMITHLAGLSDR | 0 | 1497.76 | 457.05 | 12892.55 | 103.85 |
| VDAMITHLAGLSDRR | 1 | 1653.86 | 491.77 | 4655.70 | 66.53 |
| VQLDIR | 0 | 742.43 | 0.00 | 6649.23 | 107.74 |
| YQQAVAAGQGK | 0 | 1119.57 | 31592.42 | 31575.79 | 224.70 |
| YQQAVAAGQGKAPK | 1 | 1415.75 | 14777.69 | 9049.34 | 134.08 |
|  |  |  | 288779.8 | 451419.4 |  |
